# Electrical Stimulation and Cutaneous Wound Healing: A Review of Clinical Evidence

**DOI:** 10.3390/healthcare2040445

**Published:** 2014-10-27

**Authors:** Sara Ud-Din, Ardeshir Bayat

**Affiliations:** 1 Plastic and Reconstructive Surgery Research, Manchester Institute of Biotechnology, University of Manchester, Manchester M1 7DN, UK; E-Mail: sara.ud-din@manchester.ac.uk; 2 University Hospital of South Manchester NHS Foundation Trust, Manchester Academic Health Science Centre, University of Manchester, Manchester M1 7DN, UK

**Keywords:** electrical stimulation, electrobiofeedback, wound healing, treatment, wounds, current

## Abstract

Electrical stimulation (ES) has been shown to have beneficial effects in wound healing. It is important to assess the effects of ES on cutaneous wound healing in order to ensure optimization for clinical practice. Several different applications as well as modalities of ES have been described, including direct current (DC), alternating current (AC), high-voltage pulsed current (HVPC), low-intensity direct current (LIDC) and electrobiofeedback ES. However, no one method has been advocated as the most optimal for the treatment of cutaneous wound healing. Therefore, this review aims to examine the level of evidence (LOE) for the application of different types of ES to enhance cutaneous wound healing in the skin. An extensive search was conducted to identify relevant clinical studies utilising ES for cutaneous wound healing since 1980 using PubMed, Medline and EMBASE. A total of 48 studies were evaluated and assigned LOE. All types of ES demonstrated positive effects on cutaneous wound healing in the majority of studies. However, the reported studies demonstrate contrasting differences in the parameters and types of ES application, leading to an inability to generate sufficient evidence to support any one standard therapeutic approach. Despite variations in the type of current, duration, and dosing of ES, the majority of studies showed a significant improvement in wound area reduction or accelerated wound healing compared to the standard of care or sham therapy as well as improved local perfusion. The limited number of LOE-1 trials for investigating the effects of ES in wound healing make critical evaluation and assessment somewhat difficult. Further, better-designed clinical trials are needed to improve our understanding of the optimal dosing, timing and type of ES to be used.

## 1. Introduction

Acute wounds normally undergo a complex healing process, which ultimately leads to a completely healed wound [1]. The process of acute wound healing is typically divided into a series of overlapping phases, which include: haemostasis, inflammation, proliferation, wound contraction and remodeling [2]. Normal would healing in the skin should result in the restoration of skin continuity and function. Nevertheless, there are a number of responses which can occur following a cutaneous injury; normal repair in the adult human skin should typically produce a fine line permanent scar, however, abnormal healing can result in excessive healing where there is an increased deposition of connective tissue leading to the formation of hypertrophic and keloid scars or either can deficient healing where there is insufficient deposition of connective tissue and therefore, new tissue formation is incomplete and can result in the formation of chronic wounds [2].

Chronic wounds are defined as those wounds that have failed to proceed through the reparative phases of healing in less than 42 days [3,4]. There are various factors that can delay wound healing such as diabetes, vascular insufficiency, age and nutritional deficiencies [3]. Chronic wounds represent a major health burden to both the patient and the physician and impact upon global health resources. It is estimated that the total expenditure per year in the United Kingdom for managing these wounds in the National Health Service (NHS) alone is in excess of £1bn [5,6]. The actual number of patients suffering from these wounds, are on the increase, as the ageing population and the increasing incidence of risk factors such as diabetes mellitus and smoking, result in the rising incidence of chronic wound formation. Furthermore, patients have reported that these wounds can affect their quality of life due to social isolation, reduced working hours and dependency upon the healthcare system [7].

**Figure 1:**
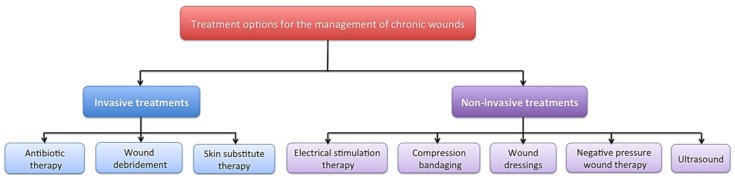
A diagram to demonstrate some of the available treatment strategies for the management of chronic wounds including; compression bandaging, wound dressings, negative pressure wound therapy, ultrasound, debridement, skin substitute therapy and electrical stimulation.

There are a range of treatment strategies available including; compression bandaging [8], wound dressings [9], negative pressure wound therapy [10], ultrasound [11], debridement [12] and skin substitutes [13], which can be expensive, time consuming and may be slow to demonstrate any positive results (Figure 1). Despite the multitude of treatment options, current regimes are not adequate, as these wounds remain a significant economic burden and a clinical problem. The use of electrical stimulation (ES) for the treatment of both acute and chronic wounds has gained prominence in the literature [14,15,16,17]. 

**Figure 2:**
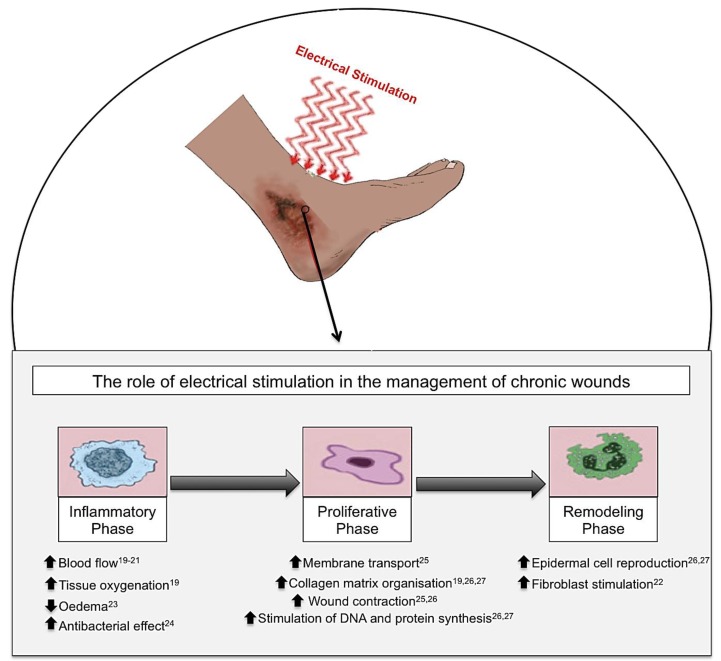
Electrical stimulation (ES), in the form of alternating current (AC), direct current (DC) and pulsed current (PC), has been shown to have beneficial effects on cutaneous wound healing in chronic wounds. When ES is applied to a chronic wound, this produces beneficial effects throughout the three phases of wound healing: inflammation, proliferation and remodelling phases. Inflammatory phase: ES increases blood flow, tissue oxygenation and stimulates fibroblasts whilst reducing oedema and providing an increased antibacterial effect. Proliferative phase: ES increases membrane transport, collagen matrix organization, wound contraction and the stimulation of DNA and protein synthesis. Remodelling phase: ES increases epidermal cell proliferation, and migration as well as stimulation of fibroblasts thus enabling enhanced wound closure [19,20,21,22,23,24,25,26,27].

**Figure 3:**
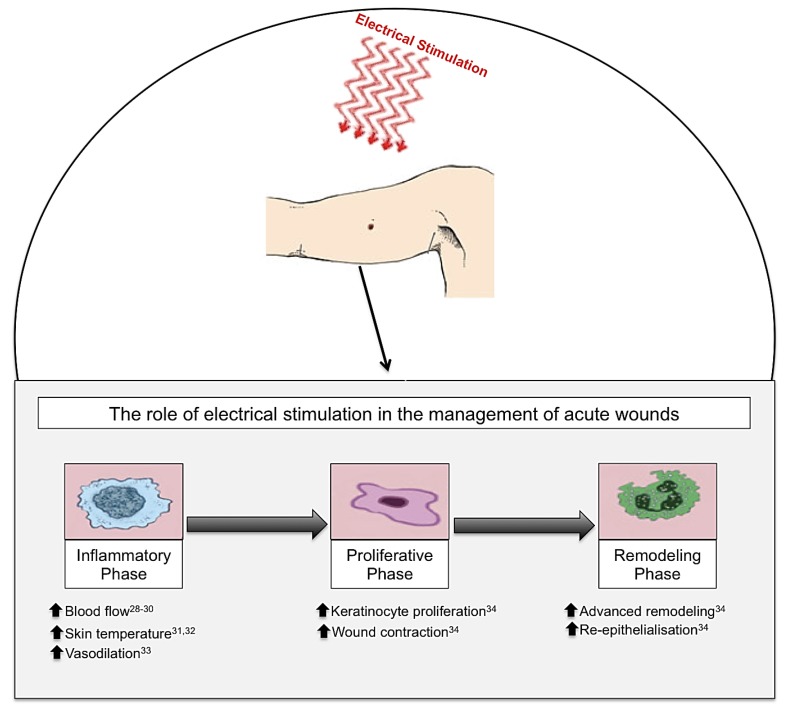
Electrical stimulation (ES), in the form of biofeedback ES, direct current (DC) and pulsed current (PC), has been shown to have beneficial effects on cutaneous wound healing in acute wounds. When ES is applied to an acute wound, this produces beneficial effects throughout the three phases of wound healing: inflammation, proliferation and remodelling phases. Inflammatory phase: ES increases blood flow, skin temperature and vasodilation. Proliferative phase: ES increases keratinocyte proliferation and wound contraction. Remodelling phase: ES advances the remodelling face and increases re-epithelialisation enabling enhanced wound healing [28,29,30,31,32,33,34].

**Figure 4:**
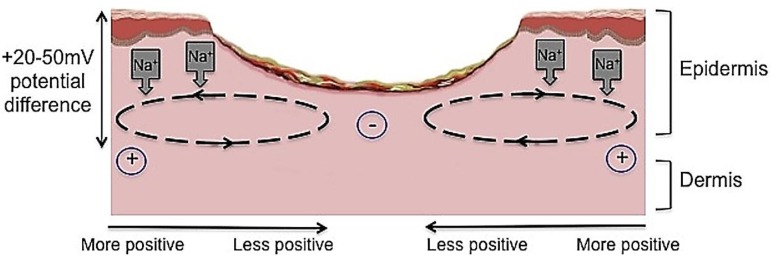
The current of injury is thought to be significant in initiating repair. Undamaged human skin has an endogenous electrical potential and a transcutaneous current potential of 20–50 mV. This is generated by the movement of sodium ions through Na+/K+ ATPase pumps in the epidermis. The current of injury is generated through epithelial disruption. Following an injury to the skin, a flow of current through the wound pathway generates a lateral electrical field and this is termed the “current of injury” or “skin battery” effect.

Many studies have advocated the use of ES therapy in conjunction with standard wound care [14,15,16,17]. ES is defined as the application of electrical current through electrodes placed on the skin either near or directly on the wound [18]. ES has been shown to have beneficial effects on the different phases of cutaneous wound healing in both chronic [19,20,21,22,23,24,25,26,27] (Figure 2) and acute wounds (Figure 3) [15,16,17,18,28,29,30,31,32,33,34]. It is suggested that ES can reduce infection, improve cellular immunity, increase perfusion, and accelerate cutaneous wound healing [35]. Undamaged human skin has an endogenous electrical potential and a transcutaneous current potential of 10–60 mV [36]. This is generated by the movement of sodium ions through Na+/K+ ATPase pumps in the epidermis [37]. Following an injury to the skin, a flow of current through the wound pathway generates a lateral electrical field and this is termed the “current of injury” or “skin battery” effect (Figure 4) [38]. Therefore, the current of injury is thought to be significant in initiating repair [38].

**Figure 5:**
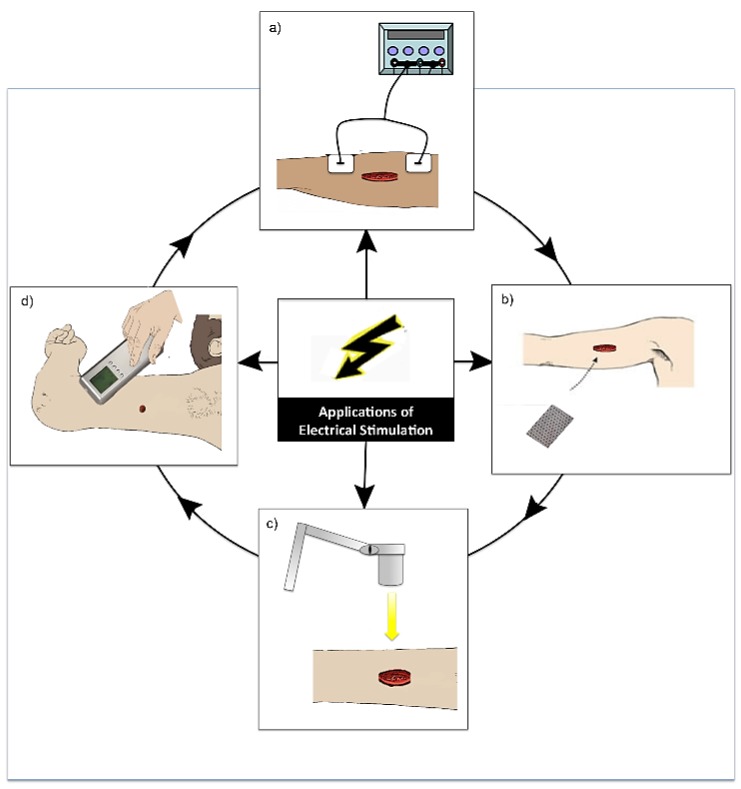
Diagram demonstrating the various modes of application of electrical stimulation (ES). (**a**) Application of ES by electrodes placed near or on the wound site and connected to a device (this is the most common application of ES) [40]; (**b**) Application of a bioelectric dressing to the wound site [41]; (**c**) Wireless application of ES to a wound [42]; (**d**) Practitioner application of ES in the form electro biofeedback by the use of a device with an electrode placed in different areas around the wound site [43].

**Figure 6:**
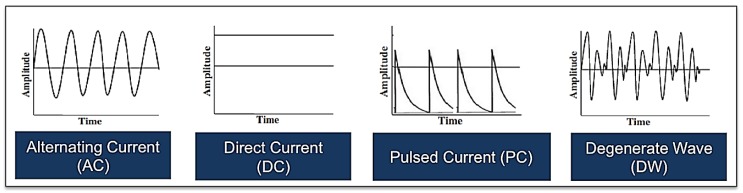
Illustrations showing one example of each of the various electrical waveforms available for the treatment of acute and chronic cutaneous wounds including alternating current, direct current, pulsed current and degenerate wave (please note that there are other subtypes of each of these waveforms).

ES has been used for a number of clinical applications, such as pain management and wound healing including chronic and acute wounds [39]. ES devices have varying voltages, currents, modes and length of time of application. Additionally, mono- or bipolar and bi or tri-electrodes are used, as well as different types of wounds indicated for each modality. There are a number of ES devices and methods of application such as dressings, electrode placement and practitioner-assisted [40,41,42,43] (Figure 5). However, the majority of trials apply the electrodes directly on the skin, and often, directly onto the wound. Several different modalities and electrical waveforms have been described (Figure 6), including direct current (DC), alternating current (AC), high-voltage pulsed current (HVPC), and low-intensity direct current (LIDC) [44]. One of the most familiar types of ES is transcutaneous electrical nerve stimulation (TENS), which has been used frequently for pain control [44,45]. Additionally, frequency rhythmic electrical modulation systems (FREMS) is also a form of transcutaneous electrotherapy using ES that varies the pulse, frequency, duration, and voltage [46]. Recently, an electrobiofeedback device, called the Fenzian system, where its waveform was found to appear as degenerate waves (DW), which degenerate over time, has been used in the treatment of acute cutaneous wound healing and reduced the symptoms associated with abnormal skin scarring [34,47,48].

Currently, there is a substantial body of work that supports the effectiveness of ES for cutaneous wound healing, although, there tends to be a poor understanding of the associated technology and its potential applications. Therefore, the aim of this review was to examine the results of clinical trials that use ES to accelerate cutaneous wound healing including the most common modalities and applications of ES. Additionally, we identified the level of evidence (LOE) supporting the use of ES in enhancing cutaneous wound healing.

## 2. Methods

**Figure 7:**
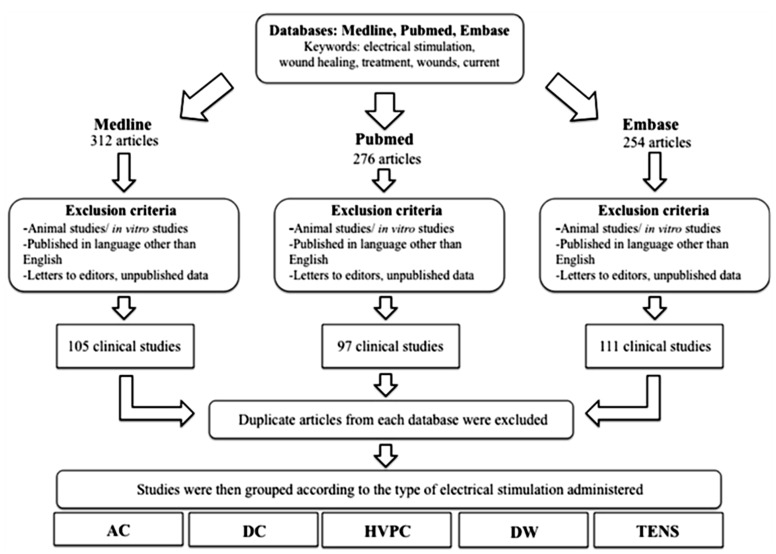
A flowchart demonstrating the methodology and process of selecting relevant articles for review.

An extensive search was conducted to identify all relevant articles published in the English language, from 1980 onwards, using the following scientific and medical search engines: PubMed, Medline and EMBASE (Figure 7). Only trials involving humans were included. Keywords used in the search included a variety of combinations such as: electrical stimulation, wound healing, treatment, wounds, electric current. All retrieved articles were reviewed for their relevance on the specific topic of electrical stimulation and cutaneous wound healing and 48 were considered suitable for inclusion in this review. Clinical studies were then grouped by the primary method of ES used and then assessed and assigned an LOE adapted from the Oxford Centre for Evidence Based Medicine to establish whether valid and reliable evidence supports the use of ES for wound healing. These levels, ranging from LOE-1 to LOE-5, are based on methodology and study design. These were assigned as follows: LOE 1 = randomized control trial; LOE-2 = cohort study; LOE-3 = case-control study; LOE-4 = Case series study; LOE-5 = expert opinion or case report.

## 3. Results

The results will now be presented under the following headings: pulsed current, direct current, transcutaneous electrical nerve stimulation, frequency rhythmic electrical modulation system, biofeedback electrical stimulation and bioelectric dressings (Table 1). Low-frequency AC has not been used successfully in the treatment of cutaneous wound healing, due to its lack of polarity [49], therefore, this modality will not be discussed.

**Table 1:** A summary table of the literature categorized under the headings; pulsed current, direct current, transcutaneous electrical nerve stimulation, frequency rhythmic electrical modulation system, biofeedback electrical stimulation and bioelectric dressings.

Author	Design	Type of Wound	Type of ES	No. Patients	Parameters	Duration	LOE Outcome
**Pulsed Current**							
**Feedar** [50]	RCT	Chronic dermal ulcers	Monophasic pulsed v sham	47	29.2 V, 29.2 mA, 132 μs, polarity reversed every 3 days then daily reversal with 64 pps	30 min twice daily for 4 weeks	1 Reduction in wound size. Wound area reduction ES 66% *vs*. sham 33% (*p* < 0.02)
**Gentzkow** [51]	Prospective	Stage III + IV pressure ulcers	Monophasic pulsed	61	128 pps, 35 mA	30 min twice daily	4 Complete healing achieved in 23%
**Baker** [52]	Prospective	Open diabetic ulcers	Asymmetric biphasic *vs*. symmetric biphasic	80	Not stated	Until ulcers healed	4 60% enhanced healing with asymmetric ES
**Franek** [53]	RCT	Pressure ulcers	High-voltage pulsed v sham	50	100 V, 100 μs, 100 Hz	50 min, once daily 5 days a week for 6 weeks	1 Improved healing rate
**Griffin** [54]	RCT	Pressure ulcers	High-voltage pulsed v sham	17	200 V, 100 pps, -ve cathode applied	1 h daily for 20 days	1 Significant increase in healing rate
**Houghton** [55]	RCT	Pressure ulcers	High-voltage pulsed v sham	34	50–100 V, 50 μs, 10–100 Hz, polarity alternated	8 h daily for 3 months	1 Improvement in wound appearance and stimulated healing with ES
**Peters** [56]	RCT	Diabetic foot ulcers	High-voltage pulsed v sham	40	50 V, 100 μs	8 h daily for 12 weeks	1 Enhanced wound healing when used with standard wound care
**Houghton** [57]	RCT	Chronic leg ulcers	High-voltage pulsed v sham	27	150 V, 100 μs, 100 Hz	3 times weekly for 4 weeks	1 Accelerated wound closure. Wound area reduction ES 44% *vs*. sham 16%
**Burdge** [58]	Retrospective	Chronic diabetic wounds	High-voltage pulsed	30	<140 V, 90–100 μs, 55.19 Hz	45 min sessions, 3 times weekly until healed approx. 16 weeks	4 Improved healing
**Goldman** [59]	RCT	Ischemic wounds	High-voltage pulsed v sham	8	100 pps, 360 V, -ve polarity	1 h daily for 14 weeks	2 Increased vasodilation and dermal capillary formation
**Ahmad** [60]	RCT	Pressure ulcers	High-voltage pulsed v sham	60	100–175 V, 50 μs, 120 Hz	Group 1: 45 min, Group 2: 60 min, Group 3: 120 min; daily for 5 weeks	1 Improved healing with ES
**Direct Current**							
**Gault** [61]	RCT	Ischemic ulcers	LIDC v sham	12	Not stated	Until healed	1 LIDC group healed twice as fast as control
**Adunsky** [59]	RCT	Pressure ulcers	DC	63	Not stated	8 weeks	1 DC useful combined with standard wound care
**Carley** [46]	Retrospective	Sacral/below knee ulcers	LIDC	30	300–500 μA for normally innervated and 500–700 μA for denervated skin	2 h, 5 days a week for 5 weeks	3 LIDC improved healing. Wound are reduction ES 89% *vs*. control 37% (*p* < 0.01)
**Wirsing** [42]	Controlled	Diabetic leg and foot ulcers	Wireless LIDC	47	1.5 μA	2–3 times weekly, 45–60 min sessions, for 8 weeks	2 Significantly accelerated healing
**Wood** [62]	Placebo controlled	Chronic decubitus ulcers	Pulsed LIDC	74	300–600 μA	8 weeks	1 Fibroblast and keratinocytes growth enhanced. Increased healing rate
**Transcutaneous Electrical Nerve Stimulation**							
**Nolan** [63]	Case study	Healthy skin	TENS	1	Not stated	20 minutes	5 Does not induce increased skin temperature
**Cramp** [29]	RCT	Over median nerve	TENS	30	High frequency: 110 Hz, 200 μsLow frequency: 4 Hz, 200 μs	15 minutes	1 No difference in skin temperature and blood flow
**Simpson** [64]	RCT	Limb ischemia	TSE	8	Not stated	1 hour daily for one week, then a week off and repeated for third week	1 No improvement in pain or microcirculation
**Cramp** [28]	RCT	Health volunteers	TENS	30	High frequency: 110 Hz, 200 μsLow frequency: 4 Hz, 200 μs	15 minutes	1 Local increase in blood flow
**Wikstrom** [65]	Controlled	Blister wound	TENS	9	High frequency: 100 Hz. Low frequency: 2 Hz	45 minutes	2 Stimulated perfusion
**Frequency Rhythmic Electrical Modulation System**							
**Jankovic** [66]	RCT	Leg ulcers	FREMS v control	35	300 V, 1000 Hz, 10–40 μs, 100–170 μA	40 min daily, 5 days a week for 3 weeks	1 Accelerated ulcer healing and reduced painWound area reduction ES 82% *vs*. control 46%
**Santamato** [67]	RCT	Venous ulcers	FREMS v control	20	Not stated	5 days a week for 3 weeks	1 Reduced pain and area of ulcers
**Biofeedback Electrical Stimulation**							
**Ud-Din** [68]	Case-series	Raised dermal scars	Biofeedback	18	0.004 mA, 20–80 V, 60 Hz	Until resolved	4 Improved scar symptoms
**Perry** [48]	Case-series	Raised dermal scars	Biofeedback	19	0.004 mA, 20–80 V, 60 Hz	Until resolved	4 Improved scar symptoms
**Ud-Din** [43]	Controlled	Acute biopsy wounds	Biofeedback	20	0.004 mA, 20–80 V, 60 Hz	2 weeks	2 Increased blood flow and haemoglobin levels
**Bioelectric Dressings**					
**Blount** [41]	Case-series	Skin graft donor sites	Bioelectric dressing	13	2–10 mV, 0.6–0.7 V, 10 μA	1 month	4 Faster healing and improved scarring
**Hampton** [69]	Case study	Leg ulcer	Bioelectric dressing	1	Not stated	Until healed	5 Improved healing
**Hampton** [70]	Case study	Pressure ulcer	Bioelectric dressing	1	Not stated	12 weeks	5 Complete healing achieved

### 3.1. Pulsed Current

Pulsed current (PC) is the unidirectional or bidirectional flow of electrons or ions, and has two waveforms: monophasic or biphasic [49]. Monophasic PC can also be described as low-voltage [52] and high voltage [53,71]. Biphasic PC is bidirectional and its waveform can be asymmetric or symmetric. PC is able to mimic the physiological endogenous current [49]. PC is delivered to the wound tissues by conductive coupling with a hydrogel or moist gauze filling the defect and the electrodes of appropriate polarity placed on top [49]. The majority of studies which used pulsed current are unidirectional. 

Low voltage PC (LVPC) devices deliver continuous DC and monophasic and biphasic waveforms of longer durations and lower voltages (20–35 V) [49]. A number of clinical studies used an LVPC device named woundEL^®^ and reported beneficial outcomes when using this for the treatment of ulcers [50,51,72]. The parameters used were: a duration of 132 microseconds and 64 pulses per second.

High-voltage pulsed current (HVPC) employs a monophasic pulsed current where the pulses are delivered in doubles. Each pulse is of short duration (less that 200 micro seconds) and it has a high peak voltage (150–500 V). HVPC is typically delivered by a device with both negative and positive electrodes either placed on the wound site or proximally on the skin [49]. This application has been used in wound healing, pain relief and oedema resolution [54,55]. A randomized controlled trial (LOE-1) conducted by Peters *et al.* studied 40 patients with diabetic foot ulcers for 12 weeks [56]. Patients were randomized to receive HVPC or sham therapy. Patients received 20 minutes of ES every hour for 8 hours each day over the 12-week study. Most patients healed in the ES group (65% compared to the sham group 35%), but the difference was not significant (*p* = 0.058). However, when patient compliance was evaluated, patients that used the device at least three times a week were more likely to heal than patients that received sham therapy and patients who used ES 0, 1, or 2 times a week (*p* = 0.038) [56].

An RCT (LOE-1) by Houghton *et al.* involved 27 patients with 42 chronic leg ulcers (arterial, venous, chronic) which were assigned to either a placebo or treatment group [57]. HVPC was delivered at 150 V, 100 pps and 100 microsecond duration. Treatments lasted for 45 minutes, 3 times a week for 4 weeks. The treatment group wounds significantly reduced in size (44%) compared to the sham group (16%). However, the significant differences were not maintained at the 1-month follow-up period. A retrospective study (LOE-4) also demonstrated positive results using HVPC in 30 patients with chronic diabetic wounds [58]. Furthermore, an RCT (LOE-1) also used this modality *versus* sham therapy in the treatment of ischemic wounds over a 14-week period and showed that the area of the wounds decreased and microcirculation was improved [59].

An RCT (LOE-1) was conducted with 60 subjects who had chronic pressure ulcers. They were split into 4 groups; one control who received sham therapy and three groups who received HVPC for 45, 60, 120 minutes respectively daily for one week [60]. Wound surface area was measured at 0, 3 and 5 weeks and they noted a significant reduction in the groups who received HVPC for 60 min and 120 min. However, no significant differences were noted between the treatment groups.

It is evident, that it is practically impossible to standardize chronic wounds in these studies, as each wound is substantially different to the next. Additionally, the research designs and device parameters were not comparable across these studies. Therefore, further larger controlled trials are critical in order to determine the optimal dosage and mode of delivery of ES.

### 3.2. Direct Current

Continuous direct current is the unidirectional flow of charged particles, which flow for 1 second or longer, and is produced by batteries, thermo couplings and solar cells [73]. The length of time the current flows has been known to cause irritation and pH changes to the skin [74]. Pulsed direct current is a monophasic pulsed waveform which flows from 1 ms to 1second [49]. Direct current is able to mimic the physiological endogenous current [49]. In wound care, a low-intensity direct current (20–1000 microamps) is used to avoid damaging healthy tissue [61]. Low-intensity direct current has been shown to promote chronic wound healing by two mechanisms: galvanotaxis (by stimulating the migration of fibroblasts and keratinocytes [75] and its antimicrobial effect [61].

A study by Adunsky *et al.* (LOE-2), 38 patients with pressure ulcers were distributed equally between shams and treatment with DC application of electrical stimulation for 8 weeks [76]. The primary outcome was percentage change in the wound area, with the results showing that wound area reduction was 31% (ES group)* vs.* 4% (sham group) (*p* = 0.09). The relatively small sample size may have contributed to the lack of significance.

Gault *et al.* conducted an 8-week trial (LOE-2) using continuous LIDC to treat 76 patients with 106 ischaemic skin ulcers [61]. They applied the negative electrode directly onto the wound for three days in order to debride necrotic tissue. The current used was 200–800 microamps for two hours, three times daily. Six patients had bilateral ulcers and therefore one ulcer was treated as a control. Forty-eight of the 100 ulcers healed completely. In the patients who had controls, healing rate for the treated ulcers was 30% compared to 14.7% in the controls. Nevertheless, a larger control group would be needed for more meaningful results. In a controlled clinical trial (LOE-2) [46], 15 unspecified wounds were treated with continuous LIDC and 15 with conservative treatment for 5 weeks. The current was 300–700 microamps for two hours in two sessions per day, five days a week. The mean healing rate for the treatment group was 89%, compared to 45% for the control group. However, limitations of this study were that despite mentioning that they had conducted a follow-up, no details were reported of this. Furthermore, the randomization process was not rigorous; participants were paired according to their age, diagnosis, wound location and aetiology with each pair placed in one of two groups. Additionally, there was no blinding as this was not possible. A recent study (LOE-3) used a wireless micro current stimulation device for the treatment of 47 patients with leg and diabetic foot ulcers [42]. This was applied 2 or 3 times a week for 60 minutes per session combined with standard wound care. They demonstrated complete healing within 3 months for the majority of cases. This device is contactless and pain-free and different wounds can be treated at the same time. 

Intermittent low-intensity direct current delivers a current, which goes up to 29.2 milliamps and then down to zero [73]. A double-blind multi-centred controlled trial (LOE-1) [62] evaluated the effect of this treatment on 43 patients with stage II and III pressure ulcers compared to 31 placebo (sham intermittent LIDC) patients. The current used was 300–600 milliamps. Twenty-five ulcers in the treatment group healed completely within 8 weeks (*p* < 0.001), compared to 4 in the control group, which had healed up to 80%. These positive results indicate a beneficial effect of intermittent LIDC, however, there was no report of randomization and no explanation for the difference in size of the two groups. Feedar *et al.* conducted a double-blind multi-centred RCT (LOE-1) using intermittent LIDC with 47 patients with 50 ulcers, which were split into control and treatment groups [50]. The current was applied at 35 milliamps, which was applied for 30 minutes, twice daily on a daily basis. They showed a statistically significant difference between the groups; the mean healing rate was 56% in the treatment group compared to 33% in the control group (*p* < 0.02).

### 3.3. Transcutaneous Electrical Nerve Stimulation

Transcutaneous Electrical Nerve Stimulation (TENS) is a low-frequency, pulsed electrical current transmitted by electrodes through the skin surface [77] to stimulate the peripheral nerves to produce various physiological effects [78]. The biphasic pulses are most commonly used [79,80]. TENS is considered to be one of the most common therapeutic modalities used in clinical practice for the relief of chronic and acute pain [78]. Some authors have observed that, in addition to its analgesic effects, TENS can also alter skin temperature and increase blood flow [40]. This observation has lead to various studies investigating the effect on the peripheral vascular system and how this facilitates tissue repair [81]. There are disagreements in the literature with regard to the increase in blood flow and skin temperature. Some studies have shown that TENS significantly increases skin temperature with low- (2 Hz to 4 Hz) [31] and high-frequency (75 Hz to 100 Hz) TENS [63], and in local blood flow [31]. However, some studies have not shown any significant increase of blood flow [29] and temperature [64] with the use of TENS. Interestingly, some studies suggested that when applied at the same intensity, low-frequency TENS enhanced blood flow levels more than high-frequency TENS [28,65].

### 3.4. Frequency Rhythmic Electrical Modulation System

Frequency rhythmic electrical modulation system (FREMS) is a form of transcutaneous electrotherapy using ES that automatically varies the pulse, frequency, duration, and voltage [46]. Two RCTs have been conducted utilising this for the treatment of chronic leg ulcers in order to improve wound healing [66,67]. The first RCT (LOE-1) recruited 35 patients and divided them into two groups [66]. One group received FREMS treatment for 2 months and the control group of no treatment. Their results showed that ulcer improvement in the treatment group was significantly higher than compared to the control group. However, a larger sample size would be needed for future studies. Another RCT (LOE-1) used FREMS treatment in 20 older patients with chronic and painful venous leg ulcers [67]. One group of 10 patients received FREMS and a topical treatment, whilst the control group received topical treatment only. 15 treatments were performed over a period of 3 weeks. They showed that there was a statistically significant decrease in ulcer area when treated with FREMS compared to the control group. Again, the small sample size means that further studies are necessary to investigate this treatment more robustly. 

### 3.5. Biofeedback ES

An electro biofeedback device termed the Fenzian system (Fenzian Ltd, Hungerford, UK), where its waveform was deciphered and shown to resemble degenerate waves, has been used successfully in the treatment of symptoms in keloid and hypertrophic scarring and in accelerating the process of acute wound healing in the skin [34,47,48,82]. It is a transcutaneous low intensity device, which detects changes in skin impedance. This device forms part of an electrobiofeedback link with the individual’s normal physiological repair. This modality follows the theory that the normal electrical potential of skin forms a global electrical network reflecting the underlying neurological activity through changes in skin impedance [82]. Using a concentric electrode the device detects the skin’s electrical impedance and adjusts the outgoing microcurrent electrical biofeedback impulses [82]. The device delivers 0.004 milliamps, 20–80 V, has a frequency default of 60 Hz and impulses which last approximately six-hundredth of a second.

It has been used successfully to alleviate the symptoms for pain, pruritus and inflammation in two case series (LOE-4) on raised dermal scars [48,68]. It is postulated that this treatment can be beneficial in the treatment of abnormal skin scarring as it may negate the need for long-term pain medications. Furthermore, a clinical trial (LOE-2) was conducted involving multiple temporal punch biopsies treated with biofeedback ES and demonstrated increased blood flow and haemoglobin levels in acute cutaneous wounds (on day 14 post-wounding) created in 20 human volunteers compared to controls which had not received ES [43]. This treatment modality accelerated the rate of cutaneous wound healing in all cases as evidenced by gene and protein studies showing up-regulated angiogenesis and down-regulated inflammation [34]. Additional larger randomised controlled trials are required to investigate this treatment further to identify if this could be beneficial in patients with chronic wounds. 

### 3.6. Bioelectric Dressings

Bioelectric dressings are emerging as a useful method of delivering ES to the wound site. However, studies of these specific modalities are lacking. Procellera^®^ is a woven metallic bandage with embedded microbatteries, which is used as a dressing for partial or full thickness wounds. The mechanism of action is delivery of ES to the wound site. It produces a low voltage of 2–10 mV by microbatteries of Ag and Zn metals which are inside a woven material and are activated by the moisture in the wound delivers 0.6–0.7 V at 10 microamps. In a study by Blount *et al.* [41], 13 patients had skin grafting and the Procellera^®^ dressing was applied to half of the donor site area. They noted improved healing, scarring and patient subjective outcomes. However, a larger trial is required to substantiate these results further. Another bioelectric wound dressing, named the PosiFect RD^®^ DC device, has been used in treating pressure and venous ulcers [69,70]. This dressing contains a miniature electrical circuit delivering a microcurrent to the wound bed for a minimum of 48 hours and has shown promise in treating these chronic wounds. 

## 4. Discussion

The reported studies demonstrate considerable variability in the parameters of ES application, leading to difficulty in generating sufficient evidence to support any one standard therapeutic approach. Most studies reported successful positive outcomes using ES to accelerate wound healing. Nonetheless, the differences in types of ulcerations or wounds, ES parameter settings and limited power of study design make synthesis of the results difficult and to draw conclusions as to an optimal mode of ES or type of ES device.

The level of evidence assigned for each study showed variations amongst the types of ES. The majority of LOE-1 were for HVPC, TENS, FREMS and DC. The available evidence (n = 4) suggests that HVPC is most beneficial for pressure ulcer treatment, with these LOE-1 studies demonstrating improved healing rates with the application of this modality. Despite a limited number of studies, HVPC has also shown positive results when used in diabetic (n = 2), ischaemic (n = 1) and chronic leg ulcers (n = 1). However, it is not apparent if other types of wounds such as acute wounds or venous ulcers would respond differently to this therapy. FREMS has been used in the treatment of leg ulcers and have shown promising results by accelerating ulcer healing and the area of the ulcer. Nevertheless, there were only two RCTs (LOE-1); thus, it is difficult to identify whether this treatment is effective in other wound types. Additionally, HVPC and DC stimulation demonstrate higher levels of evidence when compared to biofeedback ES and bioelectric dressings, which are based on case series and case study reports. There is limited clinical evidence regarding ES application for acute wounds in comparison to chronic wounds.

**Figure 8:**
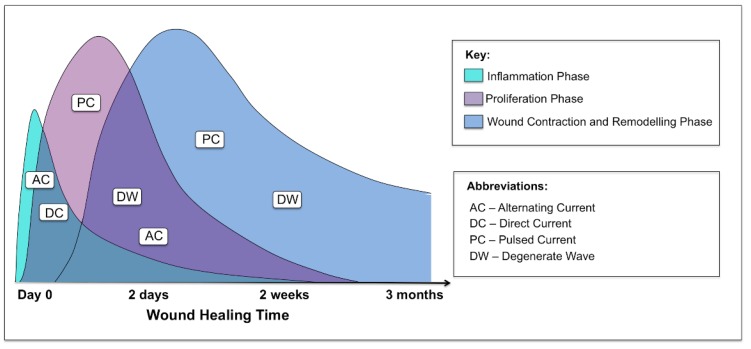
Graphical representation of the three phases of acute cutaneous wound healing and where the different waveforms of electrical stimulation are effective in each phase: inflammatory, proliferative and remodelling.

ES has mainly been evaluated in pressure ulcers, venous ulcers, vascular ulcers and diabetic foot wounds. One of the challenges in interpreting these data is the variation in outcome measurements, type of ES, and how the therapy was dosed in the trials. Most of the studies were small and many had a short treatment period and limited follow-up. In addition, many of the studies did not use complete wound healing (*i.e.*, complete wound closure) as the primary outcome. Due to the short duration of the studies, change in wound area was often used instead of wound closure. As it is difficult to standardize chronic wounds, it is important to look at acute wound studies for the effects ES has on these wounds. There was a lack of human controlled trials investigating the role of ES in acute cutaneous wounds. Biofeedback ES has been shown to be an effective method for enhancing cutaneous wound healing. A significantly increased blood flow was noted on day 14 in a controlled study [43]. Nevertheless, based on the findings to date, it remains difficult to ascertain which phases of wound healing this particular device would be optimal for. Importantly, it is of note that not all applications and modalities of ES have an effect on all phases of wound healing (Figure 8).

The majority of studies used unidirectional ES with the electrodes placed in or around the wound site. Additionally, some studies have suggested that the application of certain polarities at specific stages of wound healing may accelerate wound closure [60]. Moreover, it has been shown that electrical stimulation induces the migration of keratinocytes, which contribute to the skin’s first line of defence against pathogens, a key process in wound healing [69]. In a study by Guo *et al*, they showed that after a one-hour period the physiological electrical field enabled human dermal fibroblasts to begin migrating toward the anode, in a direction opposite to that of keratinocytes, which migrate toward the cathode [83]. This is also suggested in a study by Ahmad *et al.* [60], where they identified that applying the anode in the wound could enhance wound healing. A further study was conducted to see which was more appropriate for wound repair: anodal or cathodal microamperage direct current electrical stimulation. Application of continuous microamperage direct current is a plausible method of treatment due to the inherent potential difference between a wound and its surrounding intact skin. The study concluded that anodal microamperage direct current is more effective than cathodal microamperage direct current in healing skin wounds as it decreases the wound surface area faster, allowing for faster wound healing than cathodal electrical stimulation [84].

The majority of studies evaluated the effects of ES in patients with wounds of various aetiologies, with many having their chronic wound for a variable number of years. It is pertinent to understand when is the best time to apply ES. It may be necessary to commence treatment as soon as the wound occurs, and the exact frequency to treat with. Additionally, it may be necessary to change a chronic wound into an acute wound and then commence ES therapy [29]. When comparing the device parameters for similar wound types, it is noted that there are some variations. Pressure ulcers are a common wound type, which is used in a number of studies in particular with HVPC. Franek *et al.* [53] used parameters set at 100 V, 100 microseconds, 100 Hz for 50 minutes once daily. Griffin *et al.* [54] used a voltage of 200 V, Houghton *et al.* [55] used between 50 and 100 V and Ahmad *et al.* [60] applied 100–175 V. Therefore, the voltages applied in these studies tend to vary. Additionally, the length of time ES is to be performed is approximately similar across some studies; 50 minutes [53] and 60 minutes [54], whilst Griffin *et al.* [54] applied HVPC over a period of 8 hours per day. Ahmad *et al.* [60] compared different durations of treatment over three groups; 45 minutes, 60 minutes and 120 minutes. They noted that 60 and 120 minute groups when applied for 7 days a week for 5 weeks demonstrated optimal healing compared to the 45 minute group. In diabetic wounds, similar parameters were used for HVPC across some studies [51,71]. These studies used an interphase interval of 100 microseconds and a voltage between 50 and 140 V; however, duration of treatment times varied. Further studies comparing the parameters for different wound types and types of ES would be useful to identify the optimal settings for each device in specific wounds. 

Koel *et al.* [85] summarized the results of effect studies with ES as an additional treatment to standard wound care. They used forest plots and identified the healing rate, which was expressed as the percentage area reduction within 4 weeks of treatment. Their results showed that unidirectional ES and standard wound care increases the reduction in wound surface area by 30.8%. In pressure ulcers, the results increased to 42.7% by 4 weeks. Additionally, they noted that unidirectional ES is most beneficial for pressure ulcers, whereas venous leg ulcers and diabetic foot ulcers have had positive results with bidirectional ES. 

ES therapy is considered safe and easy to use, as no device-related complications or adverse effects have been reported to date. ES application is relatively cost effective compared to other comparative treatments. In those ES modalities which are administered by a practitioner, this can be performed by a single experienced practitioner and there is often no pain associated with the treatments. 

Some authors suggested that compliance might be a factor that affects cutaneous wound healing in ES studies [56,69]. However, in most studies, therapy was provided in a hospital or clinic setting, therefore, patients attending clinic appointments determined the main measure of compliance. The study by Peters *et al.* [56] was the only study that provided an ES device for patients to use at home and they recorded the number of hours the device was used. There was no significant difference in the compliance rates between the two treatment groups. There was a trend demonstrating a dose response with ES. A higher proportion of wounds healed in compliant patients in the ES treatment group (71%), non-compliant patients in the ES treatment group (50%), compliant patients in the sham group (39%), and non-compliant patients in the sham group (29%) [56].

In summary, despite variations in the type of current, duration, and dosing of ES, the majority of studies showed a significant improvement in wound area reduction or wound healing compared to the standard of care or sham therapy as well as improved local perfusion. Furthermore, no device-related complications or adverse effects have been reported in the existing literature, therefore, indicating that the therapy is safe and easy to use. Additionally, as ES decreases bacterial infection, increases local perfusion and accelerates wound healing, it targets these main factors of significance in wound management. There are several questions which remain unanswered, including, the optimal method of delivering ES, identifying which wound types respond better to treatment and the ideal anatomical location, frequency, duration and time to commence the application of ES for each wound type. Overall, the evidence to date infers that further clinical trials are much needed to aid in better understanding the optimal dosing, timing and type of ES to be used and to optimize the effectiveness and appropriate clinical application. No doubt, this is likely to be achieved in the future by comparing the effects of different ES modalities, treatment durations and frequencies on the rate and quality of healing in similar cutaneous wounds.
